# Factors associated with medication non-adherence among patients with heart failure*

**DOI:** 10.1590/1518-8345.6756.4302

**Published:** 2024-08-30

**Authors:** Jannaína Gomes de Lima, Alba Lucia Bottura Leite de Barros, Juliana de Lima Lopes

**Affiliations:** 1 Universidade Federal de São Paulo, Escola Paulista de Enfermagem, São Paulo, SP, Brazil.

**Keywords:** Adherence, Cardiology, Treatment Adherence and Compliance, Nursing Care, Nursing, Heart Failure

## Abstract

**Objective:**

to identify the factors contributing to medication non-adherence among patients with heart failure.

**Method:**

cross-sectional and analytical study using the *Medida de Adesão ao Tratamento* [Treatment Adherence Measure] scale to assess medication non-adherence. Independent variables were collected using the European Heart Failure Self-care Behavior Scale and an instrument developed by the authors based on a previous study. Statistical tests were implemented to analyze data with p≤0.05 statistical significance.

**Results:**

the sample comprised 340 patients, with 9.4% considered non-adherent. The multiple analysis results showed that one unit increase in an individual’s self-care score led to an 8% increase in the prevalence of non-adherence; patients with a family income above three times the minimum wage presented a prevalence of non-adherence equal to 3.5% of the prevalence of those with up to one times the minimum wage; individuals consuming alcohol or with depression presented 3.49 and 3.69 times higher prevalence of non-adherence, respectively, than individuals not presenting such history.

**Conclusion:**

medication non-adherence was associated with self-care, family income, depression, and alcohol consumption.

## Highlights

**(1)** Medication adherence was associated with self-care behaviors.

**(2)** Patients with a family income above three times the minimum wage were less likely to present medication non-adherence.

**(3)** Depression was also associated with medication non-adherence.

## Introduction

Chronic non-communicable diseases (NCDs) account for high morbidity and mortality rates worldwide^1^. These diseases include heart failure (HF), a complex clinical syndrome of a systemic nature, causing cardiac dysfunction and a blood supply that is insufficient to meet the body’s metabolic needs^2^. 

In 2021, 31,336 individuals died in Brazil due to HF^3^. It became a public health problem^4^ and accounted for 1.5 billion *Reais* costs between January 2017 and December of the same year. In the United States of America, approximately six million inhabitants aged 20 years or older were affected by HF between 2015 and 2018^5^, and an increase of approximately 46% of cases is expected in the following 15 years, i.e., more than eight million individuals are expected to be affected by HF by 2030^6^.

The treatment of HF consists of pharmacological and non-pharmacological measures and is complex in many cases^7^. The pharmacological treatment aims to alleviate symptoms and decrease morbidity, hospital readmission, and death rates due to HF^7^. The non-pharmacological treatment includes physical activity, adherence to fluid and sodium intake control, diet, smoking cessation, interruption of alcohol consumption, vaccination and monitoring of weight and signs and symptoms of HF^2^. 

Despite its relevance, adherence to self-management recommendations is suboptimal, increasing the risk of mortality and hospitalizations^8^. A multicenter study conducted in three Brazilian centers, called EMBRACE, showed that poor adherence to treatment was the leading cause of disease decompensation, representing 55% of cases. Those who reported irregular/poor adherence to treatment in the last week experienced a 22% higher risk of hospitalization^9^. An Italian study analyzed the effect of medication adherence on mortality and readmission of patients with HF. Data were collected from a database including 100,785 patients according to the number of classes of medication prescribed (one, two, or three). The results showed a 15% decrease in readmissions (OR=0.851; 95%CI=0.821-0.882; p<0.0000) among those adhering to one medication class and 29% among patients adhering to three classes of medications (OR=0.706; 95%CI=0.651-0.767; p<0.0000). Furthermore, mortality (OR=0.722; 95%CI=0.691-0.755) decreased by 28% in participants adhering to one medication class and 18% in patients adhering to three classes of medications (OR=0.818; 95%CI=0.742-0. 9; p<0.0000)^10^. Another study conducted in 47 hospitals in seven Middle Eastern countries aimed to identify the factors contributing to the readmission and mortality of patients with HF. The results showed that non-adherence to diet and medications were significant factors leading to hospital readmission and mortality (p<0.001)^11^. 

The previous discussion shows the relevance of implementing disease management programs^12^. A systematic review with meta-analysis revealed that educational interventions, telephone consultations, and home visits improve the outcomes of HF patients^13^. However, identifying the factors contributing to non-adherence is essential for nurses in planning interventions. 

Several factors impact treatment adherence. A Brazilian study conducted in Rio Grande do Sul to analyze the factors associated with poor adherence to treatment showed that non-adherence was related to advanced age, having three or more morbidities, being unable to perform instrumental activities of daily living, taking three or more medications, not having health insurance, and having to buy all or part of their medications^14^. Another national study conducted in João Pessoa (Paraíba) with 50 patients showed that those who were male, with functional class III and more than one comorbidity associated with HF obtained lower adherence scores^15^.

Considering the importance of identifying the factors associated with medication non-adherence among patients with HF for planning care and implementing interventions, and the few Brazilian studies addressing this topic, the following research question emerged: What are the factors associated with the non-adherence of patients with HF to pharmacological treatment? Therefore, this study’s objective was to identify the factors contributing to medication non-adherence among patients with heart failure.

## Method

### Study design

This cross-sectional and analytical study adopted the Strengthening the Reporting of Observational Studies in Epidemiology (STROBE) guidelines^16^.

### Setting

This study was conducted at the Cardiomyopathy Outpatient Clinic of a public hospital in São Paulo, Brazil.

### Period

Data were collected from 2018 to 2020.

### Population

The participants were patients with a medical diagnosis of HF cared for at the Cardiomyopathy Outpatient Clinic of a public hospital in São Paulo, Brazil.

### Inclusion and exclusion criteria

Patients diagnosed with HF by a medical team, older than 18 years and presenting no visual, hearing or cognitive deficits were included in the study.

### Sampling

The sample size was determined by a pilot study with 21 patients conducted from April 5 to 26, 2018. A convenient sample was selected for the pilot test, including patients who attended the service in the period previously mentioned. The sample size was based on Spearman’s Correlation Coefficient between pharmacological treatment adherence and illness duration (r= -0.162), with the significance level established at 5% and test power of 80%. Illness duration was chosen for the calculation because studies show it is an important factor contributing to treatment adherence^17^ ^)-(^ ^18^. Hence, a minimum sample of 297 participants was required; however, due to potential dropouts, the sample size was increased by 10%, and a sample of 340 patients remained. The following formula was used: $N={\left[\left(Z_{\alpha }+Z_{\beta }\right)+C\right]}^{2}+3$ , where: $C=0.5\times \ln\left[\left(1+r\right)/\left(1-r\right)\right]$ , r = expected correlation coefficient, N = Total number of subjects required, α = Significance level, and β = 1 -Test power^19^.

### Study variables

This study’s dependent variable, medication adherence, was verified by the *Medida de Adesão ao Tratamento* (MAT) [Treatment Adherence Measure]^20^. The independent variables were selected according to the literature and a previous study^21^ and then organized into sociodemographic (i.e., age, race, sex, religion, marital status, family and individual income, number of income-dependent people, employment, and educational level) and clinical variables (i.e., length of illness, number and name of prescribed medications, number of medications taken, other comorbidities, smoking, physical inactivity, alcohol intake, New York Heart Association functional class, and disease staging). The independent variable was self-care behavior, obtained with the Brazilian version of the European Heart Failure Self-care Behavior Scale - EHFScBS^22^. Data were collected through interviews and consultation on the patients’ medical records.

### Data collection

The potential participants were identified on the appointment schedule of the Cardiomyopathy Outpatient Clinic. The patients who met the inclusion criteria were personally asked whether they wanted to participate in the study. Those who voluntarily consented signed a free and informed consent form. Next, the instrument addressing the sociodemographic and clinical variables was completed. Adherence to pharmacological treatment was verified using the MAT^20^, and self-care behavior was obtained using the Brazilian version of the EHFScBS^22^.

### Data collection instruments

The authors developed the form addressing sociodemographic and clinical variables based on a previous study^21^.

The MAT^20^ was used to assess medication adherence after the authors of the original version provided their consent. The original scale was developed in Portugal, and a Cronbach’s alpha of 0.73 was obtained^20^. This scale was adapted to Brazilian Portuguese and validated among individuals with mental disorders^23^ and diabetes mellitus^24^. 

The MAT comprises seven items rated on a six-point Likert scale ranging from 1 (always) to 6 (never). Adherence is obtained by summing up each item’s score, divided by the total number of items. Individuals with scores equal to or greater than 5 were considered adherent^20^ ^),(^ ^23^ ^)-(^ ^24^.

Self-care was verified with the Brazilian version of the EHFScBS^22^. The original scale was developed and validated in 2003 by a group of researchers from the Netherlands, and a Cronbach’s alpha ranging from 0.79 to 0.92^25^ was obtained. It was translated and validated in Brazil in 2012 according to the following: translation, reconciled version, back-translation, expert panel review, pre-testing, and assessment of internal consistency (Cronbach’s alpha) and reproducibility verified through pre- and post-testing. Cronbach’s alpha ranged from 0.61 to 0.70^22^. The EHFScBS comprises five domains and 12 questions addressing self-care behavior. Alternative answers range from 1 (I strongly agree) to 5 (I strongly disagree). The total score ranges from 12 to 60: scores equal to 12 refer to the best possible self-care behavior, and scores equal to 60 refer to the worst self-care possible^22^ ^),(^ ^25^. 

### Data treatment and analysis

Quantitative variables were described by mean and standard deviation or median and quartiles, and qualitative variables were described by absolute frequency and percentage. Medication adherence was determined by two categories: adherent and non-adherent. The Mann-Whitney test was used to verify associations between medication adherence and quantitative independent variables. The Fisher’s exact test and Prevalence Ratio (PR) were used to determine the potential association with the categorical independent variables. The multiple Cox model was used to assess the joint association between independent variables and the outcome of medication adherence with constant times and robust variance. 

Variables that obtained a p-value below 0.10 in the bivariate analysis and those of clinical interest were included in the multiple analysis. The Variance Inflation Factor (VIF) was calculated to assess whether there were multicollinearity problems among the predictor variables, and no strong correlation was found. VIF values below 5 were considered the cutoff point for classifying the existence of multicollinearity^26^. R software, version 4.0^27^, was used for the statistical analyses; the statistical significance was established at p<0.05. Cronbach’s alpha coefficient measured the instrument’s internal consistency; values above 0.60 were considered acceptable^28^.

### Ethical aspects

The study project was submitted to the Institutional Review Board at Hospital São Paulo and approved on March 21, 2018 (Opinion report No. 2,555,873). According to Resolution No. 466/2012, Brazilian Health Council^29^, all participants signed a free and informed consent form.

## Results

Data were collected from 340 patients, aged 58.1±12.9 on average, who had 7.6±4.4 years of schooling on average. Regarding the duration of illness and the number of medications used, the participants had the condition for 11.8±10.3 years on average and took 6±2.2 medications on average. Most were men (51.8%), married or cohabiting (56.8%), Catholic (58.6%), of mixed race (43.78%) or Caucasian (43.2%). Additionally, most participants were unemployed (63.2%) and had a family income between one and three times the minimum wage (75.6%), followed by more than three to five times the minimum wage (11.8%). The medications most frequently used were beta-blockers (n=298; 87.6%), diuretics (n=269; 79.1%), mineralocorticoid receptor antagonists (n=236; 69.4%), statins (n= 183; 53.8%) and angiotensin-converting enzyme inhibitor or angiotensin 2 blockers (n=180; 52.9%).

The Cronbach’s alpha confirmed the instrument’s reliability (α=0.65). 

The variables in the bivariate analysis that appeared associated with treatment adherence were self-care behavior (Table 1), family income (Table 2), depression (Table 2), and the use of mineralocorticoid receptor antagonists (Table 2).

**Table 1:** Association between quantitative variables and pharmacological treatment adherence according to the Mann-Whitney test (n = 340). São Paulo, SP, Brazil, 2018-2020

Variables	Adherent (n=308)			Non-adherent (n=32)			p-value
	Median	Q25*	Q75^†^	Median	Q25*	Q75^†^	
Years of schooling	8	4	11	7	4.75	10	0.423
Self-care score	23	19	28	26.5	22	34.7	0.001
Age (complete years)	60	50	68	58.5	42	66.5	0.471
No. dependents	2	2	3	3	2	4	0.635
No. of medications taken	9	7	11	9	6	11	0.335
Duration of disease (years)	8	3	17	10	3.75	18.7	0.200

*Q25 = Quartiles 25%; ^†^Q75 = Quartiles 75%

**Table 2:** Association between qualitative sociodemographic variables and pharmacological treatment adherence according to Fisher’s exact test (n = 340). São Paulo, SP, Brazil, 2018-2020

	Adherent (n=308)	Non-adherent (n=32)	p-value	PR*	95%^†^CI
	n (%)	n (%)			
**Marital Status**					
Married/cohabiting	175 (90.7)	18 (9.3)	0.96	1	
Divorced	25 (92.6)	2 (7.4)		0.79	0.19-3.23
Single	69 (90.8)	7 (9.2)		0.99	0.43-2.27
Widowed	39 (88.6)	5 (11.4)		1.22	0.48-3.10
**Marital Status - version 2** ^ǁ^					
Married/cohabiting	175 (90.7)	18 (9.3)	1.00	1	
Others	133 (90.5)	14 (9.5)		1.02	0.52-1.98
**Race**					
Caucasian or mixed	134 (91.2)	13 (8.8)	0.843	1	
Afro-descendent	39 (88.6)	5 (11.4)		1.28	0.48-3.41
Mixed race	135 (90.6)	14 (9.4)		1.06	0.52-2.18
**Religion**					
Catholic	182 (91.5)	17 (8.5)	0.445	1	
Evangelic or others	111 (90.3)	12 (9.7)		1.14	0.56-2.30
No religion	15 (83.3)	3 (16.7)		1.95	0.63-6.03
**Family income** ^‡^					
Less than 1 time the minimum wage	9 (75)	3 (25)	0.007	1	
From 1 to 3 times the minimum wage	229 (89.1)	28 (10.9)		0.44	0.15-1.23
More than 3 times the minimum wage	69 (98.6)	1 (1.4)		0.05	0.01-0.51
Unknown	1 (100)	0 (0)		-	-
**Individual income** ^‡^					
Less than 1 time the minimum wage	36 (90)	4 (10)	0.575	1	
1 or more times the minimum wage	253 (90)	25 (10)		0.899	0.33-2.44
Does not work (unemployed or homemaker)	19 (86.4)	3 (13.6)		1.364	0.33-5.55
**Sex**					
Female	146 (89)	18 (11)	0.359	1	
Male	162 (92)	14 (8)		0.72	0.37-1.41
**Employment**					
Working	90 (90.9)	9 (9.1)	1	1	
Homemaker	24 (92.3)	2 (7.7)		0.846	0.19-3.68
Not working	194 (90.2)	21 (9.8)		1.074	0.51-2.26
**Brain Stroke**					
No	251 (90.6)	26 (9.4)	1	1	
Yes	57 (90.5)	6 (9.5)		1.015	0.44-2.36
**Arrhythmia**					
No	226 (91.1)	22 (8.9)	0.539	1	
Yes	82 (89.1)	10 (10.9)		1.22	0.60-2.49
**Asthma**					
No	295 (91.3)	28 (8.7)	0.064	1	
Yes	13 (76.5)	4 (23.5)		2.71	1.07-6.86
**Bronchitis**					
No	298 (91.1)	29 (8.9)	0.113	1	
Yes	10 (76.9)	3 (23.1)		2.60	0.91-7.45
**Functional class**					
I	71 (92.2)	6 (7.8)	0.832	1	
II	230 (89.8)	26 (10.2)		1.303	0.56-3.05
III	7 (100)	0 (0)		-	-
**Depression**					
No	286 (92.6)	23 (7.4)	0.001	1	
Yes	22 (71)	9 (29)		3.9	1.98-7.67
**Diabetes mellitus**					
No	209 (92.1)	18 (7.9)	0.236	1	
Yes	99 (87.6)	14 (12.4)		1.562	0.81-3.02
**Peripheral vascular disease**					
No	232 (90.3)	25 (9.7)	0.831	1	
Yes	76 (91.6)	7 (8.4)		0.87	0.39-1.93
**Pulmonary emphysema**					
No	300 (90.4)	32 (9.6)	1	1	
Yes	8 (100)	0 (0)		-	-
**Disease staging**					
A	72 (92.3)	6 (7.7)	0.781	1	
B	228 (89.8)	26 (10.2)		1.331	0.57-3.11
C	8 (100)	0 (0)		-	-
**Hypercholesterolemia**					
No	181 (91.9)	16 (8.1)	0.353	1	
Yes	127 (88.8)	16 (11.2)		1.38	0.71-2.66
**Hypertriglyceridemia**					
No	288 (90)	32 (10)	0.237	1	
Yes	20 (100)	0 (0)		-	-
**Systemic arterial hypertension**					
No	87 (91.6)	8 (8.4)	0.837	1	
Yes	221 (90.2)	24 (9.8)		1.163	0.54-2.49
**Alcohol consumption**					
No	295 (91.3)	28 (8.7)	0.064	1	
Yes	13 (76.5)	4 (23.5)		2.714	1.07-6.86
**Chronic kidney disease**					
No	227 (90.4)	24 (9.6)	1	1	
Yes	81 (91)	8 (9)		0.94	0.44-2.01
**Sedentariness**					
No	121 (90.3)	13 (9.7)	1	1	
Yes	187 (90.8)	19 (9.2)		0.951	0.49-1.86
**Acute coronary syndrome**					
No	213 (91)	21 (9)	0.691	1	
Yes	95 (89.6)	11 (10.4)		1.156	0.58-2.31
**Acquired immunodeficiency syndrome**					
No	307 (90.6)	32 (9.4)	1	1	
Yes	1 (100)	0 (0)		-	-
**Smoking**					
No	296 (90.5)	31 (9.5)	1	1	
Yes	12 (92.3)	1 (7.7)		0.811	0.12-5.49
**Mineralocorticoid receptor antagonists** ^§^					
No	74 (84.1)	14 (15.9)	0.017	1	
Yes	220 (93.2)	16 (6.8)		0.43	0.22-0.84
**Antiplatelet agent**					
No	211 (88.7)	27 (11.3)	0.069	1	
Yes	97 (95.1)	5 (4.9)		0.43	0.17-1.09
**Antiarrhythmic**					
No	299 (90.9)	30 (9.1)	0.277	1	
Yes	9 (81.8)	2 (18.2)		1.99	0.54-7.31
**Anticoagulant**					
No	210 (90.5)	22 (9.5)	1	1	
Yes	98 (90.7)	10 (9.3)		0.98	0.48-1.99
**Beta blocker**					
No	35 (83.3)	7 (16.7)	0.093	1	
Yes	273 (91.6)	25 (8.4)		0.50	0.23-1.09
**Calcium channel blocker**					
No	276 (91.4)	26 (8.6)	0.149	1	
Yes	32 (84.2)	6 (15.8)		1.83	0.81-4.17
**Cardiac glycosides**					
No	263 (90.4)	28 (9.6)	1	1	
Yes	45 (91.8)	4 (8.2)		0.85	0.31-2.31
**Diuretic**					
No	64 (90.1)	7 (9.9)	0.823	1	
Yes	244 (90.7)	25 (9.3)		0.94	0.42-2.09
**Statin**					
No	140 (89.2)	17 (10.8)	0.459	1	
Yes	168 (91.8)	15 (8.2)		0.76	0.39-1.47
**Oral hypoglycemic**					
No	235 (91.1)	23 (8.9)	0.664	1	
Yes	73 (89)	9 (11)		1.23	0.59-2.55
**Angiotensin 2 blocker. angiotensin-converting enzyme inhibitor or ivabradine**					
No	144 (90)	16 (10)	0.853	1	
Yes	164 (91.1)	16 (8.9)		0.89	0.46-1.72
**Insulin**					
No	292 (90.7)	30 (9.3)	0.682	1	
Yes	16 (88.9)	2 (11.1)		1.19	0.31-4.60
**Vasodilator**					
No	185 (90.7)	19 (9.3)	1	1	
Yes	123 (90.4)	13 (9.6)		1.03	0.52-2.01
**Coronary vasodilator**					
No	302 (90.7)	31 (9.3)	0.503	1	
Yes	6 (85.7)	1 (14.3)		1.53	0.24-9.71

*PR = Prevalence Ratio; ^†^CI = Confidence Interval. ^‡^Minimum Wage, Brazil, 2020 = R$ 1,039.00; ^§^Data of 16 patients were missing regarding this medication; ^ǁ^Version 2 = Dichotomized variable

Table 3 shows the Cox model’s results. Note that increasing one unit in an individual’s self-care behavior score leads to an 8% increase in non-adherence prevalence; those with a family income greater than three times the minimum wage presented a non-adherence prevalence, equal to 3.5% of the prevalence among those with up to one minimum wage. Additionally, individuals who consumed alcohol or had depression presented a non-adherence prevalence of 3.493 and 3.695 times higher, respectively, than those not consuming alcohol or experiencing depression.

**Table 3:** Results of the Cox model for pharmacological treatment adherence (n = 340). São Paulo, SP, Brazil, 2018-2020

	Cox Model*			
	VIF^†^	PR^‡^	95%^§^CI	p-value
Asthma: Yes	1.539	2.311	0.767-6.963	0.137
New York Association Functional Class	1.583	1.192	0.571-2.488	0.641
Depression: Yes	1.426	3.695	1.538-8.876	0.003
Schooling	1.781	1.007	0.913-1.111	0.884
Self-care score	1.599	1.080	1.029-1.133	0.002
Marital status: no partner	3.044	0.586	0.225-1.528	0.274
Age	1.680	0.999	0.966-1.034	0.963
Alcohol consumption: Yes	1.373	3.493	1.150-10.604	0.027
Number of medications taken	2.076	0.992	0.876-1.122	0.893
Family income: up to 3 times the MW	2.269	0.270	0.071-1.024	0.054
Family income: more than 3 times the MW	2.269	0.035	0.004-0.328	0.003
Disease duration in years	1.963	1.011	0.982-1.042	0.453

*Cox = Cox Model; ^†^VIF = Variance Inflation Factor; ^‡^PR = Prevalence Ratio; ^§^CI = Confidence Interval

## Discussion

Medication adherence is influenced by several behavioral, social and economic factors, and monitoring such factors is essential to ensure treatment success^30^.

This study’s results show that most participants adhered to the pharmacological treatment, corroborating the literature findings. A cross-sectional study was conducted in São Paulo, with 100 patients with HF and showed that more than half of the participants were adherent or moderately adhered to the treatment^31^. The results of a study in Thailand addressing 180 patients with HF indicated that 11.7% of the participants presented low medication adherence^32^. 

The result concerning medication adherence for most participants might be related to the fact that HF is a chronic disease in which individuals must adapt to a new lifestyle and regularly take many medications to prevent decompensation and preserve their routine and quality of life^32^.

Although most patients adhered to the pharmacological treatment, four factors emerged in this study related to non-adherence. The association between worse self-care behavior and non-adherence was expected. According to the World Health Organization (WHO), self-care is defined as “the ability of individuals, families, and communities to promote and maintain their own health, prevent disease, and to cope with illness - with or without the support of a health or care worker”^33^. Furthermore, the correct use of medications is one of the components of self-care^30^. The literature reports self-care to be cardio protective and complement pharmacological and clinical treatments with the potential to delay the progression of HF and its undesirable results, such as clinical decompensation and hospital readmissions^34^. The findings of a study conducted in western Ethiopia corroborate the results found here. This Ethiopian study addressed 424 individuals with chronic heart failure and found that more than half of the participants adhered to the pharmacological treatment. Additionally, those with adequate adherence were more likely to present improved self-care behaviors (OR=4.214; 95%CI=2.725-6.515; p<0.001)^35^.

Low family income has also been identified in the literature as one of the factors for medication non-adherence. A study addressing 142,577 individuals with chronic cardiovascular diseases sought to identify the sociodemographic factors associated with medication non-adherence and found that low income was related to non-adherence (OR=3.57; 95% CI= 2.11-6.02)^36^. One of the reasons is not having enough money to buy the medications not provided by the Brazilian Unified Health System, besides the costs of accessing health services.

This study’s results show two other variables associated with medication non-adherence: depression and alcohol consumption. Depression has been associated with decreased adherence to medication treatment and lack of ability and/or interest in self-care, resulting in poorer quality of life, higher expenses with health services, and increased mortality rates^37^ ^)-(^ ^38^. As for alcohol consumption, a study suggests that alcohol may not be directly related to medication non-adherence but rather to the fact that it triggers other physical and mental health problems that worsen sleep quality, constituting factors associated with non-adherence^39^. Another potential explanation for such a relationship includes the possibility of patients forgetting to take their medications due to the effects of alcohol^40^, being afraid of potential interactions between medications and alcohol and lack of money to buy medications due to alcohol consumption^40^.

Limitations include the fact that it is a cross-sectional study, which hinders the establishment of causal relationships. Additionally, it was conducted in the outpatient clinic of a single center, so the results cannot be generalized to individuals with different characteristics or hospitalized. The instrument adopted here was not submitted for analysis of psychometric properties in previous studies considering this population, and the internal consistency of the instrument used to verify treatment adherence was considered acceptable. Therefore, studies with larger samples assessing MAT’s performance and psychometric properties among individuals with HF are needed.

Despite the limitations previously discussed, this study’s results present relevant implications for clinical practice. It is essential to acknowledge that individuals with HF presenting worse self-care behavior, low family incomes, depression, or consuming alcohol are more likely to fail to adhere to the pharmacological treatment. Therefore, individuals with these characteristics require better management throughout their clinical trajectory. For this reason, nurses must be aware of these factors to plan interventions. Nurses are among the professionals who contribute to health education the most, especially among individuals with chronic diseases, such as HF. Thus, this study’s findings are expected to contribute substantially to interventions so that individuals presenting the same clinical and sociodemographic characteristics found here obtain the best outcomes possible.

Therefore, considering the few Brazilian studies in the field, further research is needed to identify and analyze interventions that can effectively improve medication adherence and implement them in clinical practice to improve the quality of life of patients with HF and decrease hospital readmissions and deaths.

## Conclusion

Most participants adhered to the pharmacological treatment. The factors associated with non-adherence were inadequate self-care behavior, lower family income, depression, and alcohol intake.
