# Supplementary material for: Factors associated with medication non-adherence among patients with heart failure
Source: Rev Lat Am Enfermagem. 2024 Aug 30;32:e4302. doi: 10.1590/1518-8345.6756.4302 (PMC11368070; doi:10.1590/1518-8345.6756.4302)
Supplement: Translation

# Translation: Fatores associados à não adesão ao tratamento farmacológico de pacientes com insuficiência cardíaca*

**DOI:** 10.1590/1518-8345.6756.4303

**Authors:** Jannaína Gomes de Lima, Alba Lucia Bottura Leite de Barros, Juliana de Lima Lopes

**Affiliations:** 1 Universidade Federal de São Paulo, Escola Paulista de Enfermagem, São Paulo, SP, Brasil.

**Keywords:** Adesão à Medicação, Cardiologia, Cooperação e Adesão ao Tratamento, Cuidados de Enfermagem, Enfermagem, Insuficiência Cardíaca

## Abstract

**Objetivo:**

identificar os fatores que contribuem para a não adesão ao tratamento farmacológico de pacientes com insuficiência cardíaca.

**Método:**

estudo transversal e analítico que utilizou a escala de Medida de Adesão ao Tratamento para avaliar a não adesão ao tratamento farmacológico. Variáveis independentes foram coletadas utilizando-se a *European Heart Failure Self-care Behavior Scale* e um instrumento elaborado pelos autores, baseado em estudo anterior. Foram utilizados testes estatísticos para análise dos dados, sendo considerados significativos os valores de p≤0,05.

**Resultados:**

a amostra foi composta por 340 pacientes. Desses, 9,4% foram classificados como não aderentes. Os resultados da análise múltipla mostraram que o aumento de uma unidade no escore de autocuidado leva a um aumento de 8% na prevalência de não adesão do indivíduo; pacientes com renda familiar superior a três salários mínimos têm prevalência de não adesão ao tratamento igual a 3,5% da prevalência entre aqueles com até um salário mínimo; indivíduos que ingerem bebida alcoólica e sofrem de depressão têm prevalências de não adesão 3,49 e 3,69 vezes maiores, respectivamente, do que aqueles que não têm tais antecedentes.

**Conclusão:**

a não adesão ao tratamento farmacológico relacionou-se com comportamento de autocuidado, renda familiar, depressão e ingestão de bebida alcoólica.

## Destaques

**(1)** A adesão ao tratamento farmacológico relacionou-se com o comportamento de autocuidado.

**(2)** Pacientes com renda familiar maior que três salários têm menor propensão à não adesão.

**(3)** Outra associação com a não adesão ao tratamento farmacológico foi a depressão.

## Introdução

As doenças crônicas não transmissíveis (DCNT) são responsáveis por elevadas taxas de morbidade e de mortalidade em todo o mundo^1^. Dentre essas doenças, inclui-se a insuficiência cardíaca (IC), definida como uma síndrome clínica complexa de caráter sistêmico, ocasionando disfunção cardíaca e inadequado suprimento sanguíneo para atender às necessidades metabólicas do organismo^2^. 

No Brasil, no ano de 2021, foi constatado que 31.336 indivíduos morreram em decorrência da IC^3^ e que essa doença foi responsável por um custo de 1,5 bilhão de reais entre janeiro de 2017 e dezembro daquele ano, tornando-se um problema de saúde pública^4^. Por sua vez, nos Estados Unidos da América, aproximadamente seis milhões de habitantes com idade maior ou igual a 20 anos tinham IC entre 2015 e 2018^5^, com um aumento estimado em aproximadamente 46% de casos nos 15 anos seguintes, perfazendo mais de oito milhões de indivíduos com IC até 2030^6^.

O tratamento da IC é composto por medidas farmacológicas e não farmacológicas. Em muitos casos, esse tratamento é complexo^7^. O tratamento farmacológico tem como finalidade amenizar os sintomas e reduzir a morbidade e as taxas de readmissões e de óbitos decorrentes da IC^7^. Por sua vez, o tratamento não farmacológico enfoca atividade física, adesão ao controle da ingestão de líquidos e sódio, cuidados com a alimentação, cessação do tabaco, interrupção do uso de álcool, vacinação e monitorização do peso e dos sinais e sintomas da IC^2^. 

Apesar de sua importância, a adesão às recomendações de autogerenciamento da saúde está abaixo do ideal, o que aumenta o risco de mortalidade e hospitalizações^8^. Estudo multicêntrico realizado em três centros brasileiros, denominado EMBRACE, mostrou que a má adesão ao tratamento foi o principal motivo de descompensação da doença, representando 55% dos casos. Por sua vez, os que referiram uso irregular na última semana apresentaram um risco 22% maior de internação por má adesão^9^. Pesquisa desenvolvida na Itália analisou o efeito da adesão ao tratamento medicamentoso na mortalidade e na readmissão de pacientes com IC. Os dados dessa pesquisa foram extraídos de um banco de dados que analisou 100.785 pacientes, que foram agrupados com base no número de classes medicamentosas prescritas (uma, duas ou três). Os resultados mostraram que houve redução de 15% de readmissões (OR=0,851; IC95%=0,821-0,882; p<0,0000) em pacientes aderentes a uma classe medicamentosa e de 29% em pacientes aderentes a três classes (OR=0,706; IC95%=0,651-0,767; p<0,0000). Ainda, houve redução de 28% na mortalidade (OR=0,722; IC95%=0,691-0,755) de participantes aderentes a uma classe medicamentosa e de 18% nos pacientes aderentes a três classes (OR=0,818; IC95%=0,742-0,9; p<0,0000)^10^. Outro estudo conduzido em 47 hospitais de sete países do Oriente Médio teve como objetivo identificar os fatores que contribuem para a readmissão e a mortalidade de pacientes com IC. Os resultados encontrados apontaram que a não adesão à dieta e a não adesão aos medicamentos são fatores precipitantes e significativos na readmissão e na mortalidade (p<0,001)^11^. 

Diante desse contexto, a implementação de programas de manejo da doença é de extrema importância^12^. Uma revisão sistemática com meta-análise evidenciou que intervenções educativas, consulta telefônica e visita domiciliar contribuem para a melhora do desfecho dos pacientes com IC^13^. Entretanto, torna-se necessário identificar os fatores que contribuem para a não adesão, de modo que os enfermeiros possam direcionar as suas intervenções.

Há diversos fatores que podem impactar a adesão ao tratamento. Pesquisa brasileira realizada no Rio Grande do Sul, cujo objetivo era analisar quais os fatores associados à má adesão ao tratamento, apresentou, em seus resultados, que a não adesão estava relacionada à idade avançada, ter três ou mais morbidades, possuir incapacidade instrumental para vida diária, tomar três ou mais medicamentos, não ter plano de saúde e ter que comprar todos ou parte dos medicamentos^14^. Outro estudo nacional realizado em João Pessoa (Paraíba), com 50 pacientes, evidenciou que aqueles do sexo masculino, com classe funcional III e com mais de uma comorbidade associada à IC demonstraram menores escores de adesão^15^.

Considerando a importância da identificação dos fatores associados à não adesão ao tratamento medicamentoso dos pacientes com IC para o planejamento da assistência e a implementação de intervenções, bem como a escassez de publicações no Brasil, surgiu a seguinte questão de pesquisa: quais são os fatores que se relacionam com a não adesão ao tratamento farmacológico de pacientes com IC? Dessa forma, o objetivo deste estudo foi identificar os fatores que contribuem para a não adesão ao tratamento farmacológico de pacientes com insuficiência cardíaca. 

## Método

### Tipo do estudo

Trata-se de um estudo transversal e analítico. Para o desenvolvimento desta seção, utilizamos as diretrizes recomendadas pelo *Strengthening the Reporting of Observational Studies in Epidemiology* (STROBE)^16^.

### Local

O estudo foi realizado no Ambulatório de Miocardiopatia de um hospital público do Estado de São Paulo.

### Período

A coleta de dados ocorreu no período de 2018 a 2020.

### População

Os participantes da pesquisa foram pacientes com diagnóstico médico de IC atendidos no Ambulatório de Miocardiopatia de um hospital público do estado de São Paulo.

### Critérios de inclusão e exclusão

Pacientes com IC, maiores de 18 anos, sem déficit visual, auditivo e/ou cognitivo, diagnosticados pela equipe médica. 

### Definição da amostra

O tamanho da amostra foi determinado por um estudo-piloto com 21 pacientes, realizado no período de 5 a 26 de abril de 2018. A amostra do teste-piloto foi por conveniência, inserindo os pacientes que apareceram no serviço naquele período. O cálculo do tamanho amostral foi baseado no Coeficiente de Correlação de Spearman entre a adesão ao tratamento medicamentoso e o tempo de doença (r= -0,162), com nível de significância de 5% e poder de teste de 80%. A escolha do tempo de doença para o cálculo deve-se ao fato de que estudos mostram que o tempo de doença é um importante fator que contribui para a adesão ao tratamento^17^ ^)-(^ ^18^. A partir desse cálculo, obteve-se uma amostra mínima de 297 participantes. Entretanto, devido a possíveis desistências, foi aumentado o tamanho da amostra em 10%, ou seja, 340 pacientes. Para a realização desse cálculo, utilizamos a seguinte fórmula: $N={\left[\left(Z_{\alpha }+Z_{\beta }\right)+C\right]}^{2}+3$ , onde: $C=0.5\times \ln\left[\left(1+r\right)/\left(1-r\right)\right]$ , r = coeficiente de correlação esperado, N = Número total de sujeitos necessários, α = Nível de significância e β = 1 - Poder do teste^19^. 

### Variáveis do estudo

A variável dependente do estudo foi a adesão ao tratamento medicamentoso, obtida pela escala de Medida de Adesão ao Tratamento (MAT)^20^. As variáveis independentes foram selecionadas de acordo com estudos da literatura e pesquisa anterior^21^. Em seguida, foram divididas em sociodemográficas (idade, raça, sexo, religião, estado civil, renda familiar e individual, número de pessoas dependentes da renda, vínculo empregatício e escolaridade) e clínicas (tempo da doença, número e nome dos medicamentos prescritos, número de tomadas dos medicamentos, presença de outras comorbidades, tabagismo, sedentarismo, ingestão de bebida alcoólica, classe funcional da *New York Heart Association* e estadiamento da doença). Outra variável independente investigada foi o comportamento de autocuidado, obtido por meio da versão brasileira da *European Heart Failure Self-care Behavior Scale* - EHFScBS^22^. Essas variáveis foram obtidas por meio de entrevista e/ou consulta ao prontuário do paciente.

### Procedimentos para a coleta de dados

Os potenciais participantes do estudo foram identificados a partir da agenda de consultas do Ambulatório de Miocardiopatia. Os pacientes que atenderam aos critérios de inclusão foram consultados pessoalmente quanto ao desejo em participar da pesquisa voluntariamente. Aqueles que concordaram assinaram o Termo de Consentimento Livre e Esclarecido. Posteriormente, foi preenchido o instrumento que continha as variáveis sociodemográficas e clínicas. A adesão ao tratamento medicamentoso foi avaliada por meio da escala da MAT^20^. O comportamento de autocuidado foi obtido por meio da versão brasileira da EHFScBS^22^.

### Instrumentos para a coleta de dados

O instrumento contendo as variáveis sociodemográficas e clínicas foi elaborado pelos autores do presente estudo e baseado em estudo anterior^21^.

A adesão ao tratamento medicamentoso foi avaliada por meio da escala MAT^20^, após autorização dos autores da escala original. A escala original foi desenvolvida em Portugal e apresenta um valor de alfa de Cronbach de 0,73^20^. No Brasil, essa escala foi traduzida e validada para a língua portuguesa em indivíduos com transtorno mental^23^ e com diabetes mellitus^24^. 

Esse questionário é formado por sete itens e por uma escala do tipo Likert de seis pontos que varia de 1 (sempre) a 6 (nunca). O nível de adesão é obtido somando-se o valor de cada item e dividindo-se pelo número total de itens. Assim, os indivíduos que obtiverem escores igual ou superior a 5 são considerados como aderentes^20^ ^),(^ ^23^ ^)-(^ ^24^.

O comportamento de autocuidado foi obtido por meio da versão brasileira da EHFScBS^22^. A escala original foi desenvolvida e validada em 2003 por um grupo de pesquisadores da Holanda. O alfa de Cronbach variou de 0,79 a 0,92^25^. Foi traduzida e validada no Brasil em 2012, quando foram realizadas as etapas de tradução, síntese, retrotradução, revisão por Comitê de Especialistas, pré-teste, avaliação de consistência interna (alfa de Cronbach) e reprodutibilidade por meio do pré- e pós-teste. O alfa de Cronbach variou de 0,61 a 0,70^22^. A EHFScBS possui cinco domínios e doze questões relacionadas ao comportamento de autocuidado. As respostas para cada item variam de 1 (eu concordo plenamente) a 5 (eu discordo plenamente). A pontuação varia de 12 a 60 pontos, em que 12 é o melhor autocuidado possível e 60 é o pior autocuidado^22^ ^),(^ ^25^.

### Tratamento e análises de dados

As variáveis quantitativas foram descritas por média e desvio-padrão ou mediana e quartis, enquanto as qualitativas o foram por frequência absoluta e porcentagem. A adesão ao tratamento farmacológico foi avaliada considerando duas categorias: aderente e não aderente. Para verificar a associação da adesão ao tratamento farmacológico com as variáveis independentes quantitativas, foi utilizado o teste de Mann-Whitney. Para a associação com as variáveis independentes categóricas, utilizou-se o teste exato de Fisher e a medida de Razão de Prevalência (RP). Para avaliar a associação conjunta entre diferentes variáveis independentes e o desfecho adesão ao tratamento farmacológico, foi utilizado o modelo múltiplo de Cox, com tempos constantes e variância robusta.

As variáveis que obtiveram um p-valor menor do que 0,10 na análise bivariada e as de interesse clínico para os pesquisadores foram incluídas na análise múltipla. Para avaliar se havia problemas de multicolinearidade entre as variáveis preditoras, foi calculada a métrica *Variance Inflation Factor* (VIF), a qual indicou que não havia forte correlação entre tais variáveis. Valores de VIF inferiores a 5 foram considerados como corte para classificação da existência ou não de multicolinearidade^26^. Foi utilizado o *software* estatístico R, versão 4.0^27^, para as análises estatísticas, sendo consideradas estatisticamente relevantes as que obtiveram valor de p≤0,05. A consistência interna do instrumento foi medida pelo coeficiente alfa de Cronbach, sendo considerados aceitáveis valores acima de 0,60^28^.

### Aspectos éticos

O projeto de pesquisa foi submetido ao Comitê de Ética em Pesquisa do Hospital São Paulo, sendo aprovado no dia 21 de março de 2018, sob o Parecer nº 2.555.873. Todos os participantes assinaram o Termo de Consentimento Livre e Esclarecido, atendendo à Resolução n.º 466/2012 do Conselho Nacional de Saúde^29^.

## Resultados

Foram coletados os dados de 340 pacientes, com idade média de 58,1±12,9 anos, os quais possuíam, em média, 7,6±4,4 anos de estudos. Ao analisar o tempo de doença e a quantidade de medicamentos utilizados, observou-se que os participantes apresentavam, em média, 11,8±10,3 anos de tempo da doença e utilizavam, em média, 6±2,2 medicamentos. A maioria consistia em indivíduos do sexo masculino (51,8%), casados ou amasiados (56,8%) e da religião católica (58,6%), sendo predominantemente pardos (43,78%) e brancos (43,2%). Observou-se que a maioria não possuía vínculo empregatício (63,2%), com renda familiar entre um e três salários mínimos (75,6%), seguida de mais de três até cinco salários mínimos (11,8%).

Os medicamentos mais utilizados foram os betabloqueadores (n=298; 87,6%), diuréticos (n=269; 79,1%), antagonistas dos receptores do mineralocorticosteroide (n=236; 69,4%), estatina (n= 183; 53,8%) e inibidor da enzima conversora da angiotensina ou bloqueador da angiotensina 2 (n=180; 52,9%).

Os resultados do alfa de Cronbach indicaram uma confiabilidade aceitável do instrumento (α=0,65).

Na análise bivariada, as variáveis que se relacionaram com a adesão ao tratamento foram: comportamento de autocuidado (Tabela 1), renda familiar (Tabela 2), depressão (Tabela 2) e uso de antagonistas dos receptores de mineralocorticoides (Tabela 2). 

**Tabela 1:** Relação das variáveis quantitativas com a adesão ao tratamento farmacológico de acordo com o teste de Mann-Whitney (n = 340). São Paulo, SP, Brasil, 2018-2020

Variáveis	Aderente (n=308)			Não aderente (n=32)			p-valor
	Mediana	Q25*	Q75^†^	Mediana	Q25*	Q75^†^	
Escolaridade	8	4	11	7	4,75	10	0,423
Escore de autocuidado	23	19	28	26,5	22	34,7	0,001
Idade (anos completos)	60	50	68	58,5	42	66,5	0,471
Número de pessoas dependentes da renda	2	2	3	3	2	4	0,635
Número de tomadas de medicamentos	9	7	11	9	6	11	0,335
Tempo da doença em anos	8	3	17	10	3,75	18,7	0,200

*Q25 = Quartis 25%; ^†^Q75 = Quartis 75%

**Tabela 2:** Relação das variáveis sociodemográficas qualitativas com a adesão ao tratamento farmacológico de acordo com o teste exato de Fisher (n = 340). São Paulo, SP, Brasil, 2018-2020

	Aderente (n=308)	Não aderente (n=32)	p-valor	RP*	IC^†^ 95%
	n (%)	n (%)			
**Estado civil**					
Casado/amasiado	175 (90,7)	18 (9,3)	0,96	1	
Divorciado	25 (92,6)	2 (7,4)		0,79	0,19-3,23
Solteiro	69 (90,8)	7 (9,2)		0,99	0,43-2,27
Viúvo	39 (88,6)	5 (11,4)		1,22	0,48-3,10
**Estado civil - versão 2** ^ǁ^					
Casado/amasiado	175 (90,7)	18 (9,3)	1,00	1	
Outros	133 (90,5)	14 (9,5)		1,02	0,52-1,98
**Raça**					
Branca ou mestiça	134 (91,2)	13 (8,8)	0,843	1	
Negra	39 (88,6)	5 (11,4)		1,28	0,48-3,41
Parda	135 (90,6)	14 (9,4)		1,06	0,52-2,18
**Religião**					
Católico	182 (91,5)	17 (8,5)	0,445	1	
Evangélico e outras religiões	111 (90,3)	12 (9,7)		1,14	0,56-2,30
Sem religião	15 (83,3)	3 (16,7)		1,95	0,63-6,03
**Renda familiar** ^‡^					
Menos de um salário mínimo	9 (75)	3 (25)	0,007	1	
De um até três salários mínimos	229 (89,1)	28 (10,9)		0,44	0,15-1,23
Mais de três salários mínimos	69 (98,6)	1 (1,4)		0,05	0,01-0,51
Não sabe	1 (100)	0 (0)		-	-
**Renda individual** ^‡^					
Menos de um salário mínimo	36 (90)	4 (10)	0,575	1	
Um salário mínimo ou mais	253 (90)	25 (10)		0,899	0,33-2,44
Não trabalha (desempregado ou do lar)	19 (86,4)	3 (13,6)		1,364	0,33-5,55
**Sexo**					
Feminino	146 (89)	18 (11)	0,359	1	
Masculino	162 (92)	14 (8)		0,72	0,37-1,41
**Vínculo empregatício**					
Ativo	90 (90,9)	9 (9,1)	1	1	
Do lar	24 (92,3)	2 (7,7)		0,846	0,19-3,68
Inativo	194 (90,2)	21 (9,8)		1,074	0,51-2,26
**Acidente vascular encefálico**					
Não	251 (90,6)	26 (9,4)	1	1	
Sim	57 (90,5)	6 (9,5)		1,015	0,44-2,36
**Arritmia**					
Não	226 (91,1)	22 (8,9)	0,539	1	
Sim	82 (89,1)	10 (10,9)		1,22	0,60-2,49
**Asma**					
Não	295 (91,3)	28 (8,7)	0,064	1	
Sim	13 (76,5)	4 (23,5)		2,71	1,07-6,86
**Bronquite**					
Não	298 (91,1)	29 (8,9)	0,113	1	
Sim	10 (76,9)	3 (23,1)		2,60	0,91-7,45
**Classe funcional**					
I	71 (92,2)	6 (7,8)	0,832	1	
II	230 (89,8)	26 (10,2)		1,303	0,56-3,05
III	7 (100)	0 (0)		-	-
**Depressão**					
Não	286 (92,6)	23 (7,4)	0,001	1	
Sim	22 (71)	9 (29)		3,9	1,98-7,67
**Diabetes mellitus**					
Não	209 (92,1)	18 (7,9)	0,236	1	
Sim	99 (87,6)	14 (12,4)		1,562	0,81-3,02
**Doença vascular periférica**					
Não	232 (90,3)	25 (9,7)	0,831	1	
Sim	76 (91,6)	7 (8,4)		0,87	0,39-1,93
**Enfisema pulmonar**					
Não	300 (90,4)	32 (9,6)	1	1	
Sim	8 (100)	0 (0)		-	-
**Estadiamento da doença**					
A	72 (92,3)	6 (7,7)	0,781	1	
B	228 (89,8)	26 (10,2)		1,331	0,57-3,11
C	8 (100)	0 (0)		-	-
**Hipercolesterolemia**					
Não	181 (91,9)	16 (8,1)	0,353	1	
Sim	127 (88,8)	16 (11,2)		1,38	0,71-2,66
**Hipertrigliceridemia**					
Não	288 (90)	32 (10)	0,237	1	
Sim	20 (100)	0 (0)		-	-
**Hipertensão arterial sistêmica**					
Não	87 (91,6)	8 (8,4)	0,837	1	
Sim	221 (90,2)	24 (9,8)		1,163	0,54-2,49
**Ingestão de bebida alcoólica**					
Não	295 (91,3)	28 (8,7)	0,064	1	
Sim	13 (76,5)	4 (23,5)		2,714	1,07-6,86
**Insuficiência renal crônica**					
Não	227 (90,4)	24 (9,6)	1	1	
Sim	81 (91)	8 (9)		0,94	0,44-2,01
**Sedentarismo**					
Não	121 (90,3)	13 (9,7)	1	1	
Sim	187 (90,8)	19 (9,2)		0,951	0,49-1,86
**Síndrome coronariana aguda**					
Não	213 (91)	21 (9)	0,691	1	
Sim	95 (89,6)	11 (10,4)		1,156	0,58-2,31
**Síndrome da imunodeficiência adquirida**					
Não	307 (90,6)	32 (9,4)	1	1	
Sim	1 (100)	0 (0)		-	-
**Tabagismo**					
Não	296 (90,5)	31 (9,5)	1	1	
Sim	12 (92,3)	1 (7,7)		0,811	0,12-5,49
**Antagonistas dos receptores de mineralocorticoides** ^§^					
Não	74 (84,1)	14 (15,9)	0,017	1	
Sim	220 (93,2)	16 (6,8)		0,43	0,22-0,84
**Antiagregante plaquetário**					
Não	211 (88,7)	27 (11,3)	0,069	1	
Sim	97 (95,1)	5 (4,9)		0,43	0,17-1,09
**Antiarrítmico**					
Não	299 (90,9)	30 (9,1)	0,277	1	
Sim	9 (81,8)	2 (18,2)		1,99	0,54-7,31
**Anticoagulante**					
Não	210 (90,5)	22 (9,5)	1	1	
Sim	98 (90,7)	10 (9,3)		0,98	0,48-1,99
**Betabloqueador**					
Não	35 (83,3)	7 (16,7)	0,093	1	
Sim	273 (91,6)	25 (8,4)		0,50	0,23-1,09
**Bloqueador do canal de cálcio**					
Não	276 (91,4)	26 (8,6)	0,149	1	
Sim	32 (84,2)	6 (15,8)		1,83	0,81-4,17
**Digitálico**					
Não	263 (90,4)	28 (9,6)	1	1	
Sim	45 (91,8)	4 (8,2)		0,85	0,31-2,31
**Diurético**					
Não	64 (90,1)	7 (9,9)	0,823	1	
Sim	244 (90,7)	25 (9,3)		0,94	0,42-2,09
**Estatina**					
Não	140 (89,2)	17 (10,8)	0,459	1	
Sim	168 (91,8)	15 (8,2)		0,76	0,39-1,47
**Hipoglicemiante oral**					
Não	235 (91,1)	23 (8,9)	0,664	1	
Sim	73 (89)	9 (11)		1,23	0,59-2,55
**Bloqueador angiotensina 2, inibidor da enzima conversora da angiotensina ou ivabradina**					
Não	144 (90)	16 (10)	0,853	1	
Sim	164 (91,1)	16 (8,9)		0,89	0,46-1,72
**Insulina**					
Não	292 (90,7)	30 (9,3)	0,682	1	
Sim	16 (88,9)	2 (11,1)		1,19	0,31-4,60
**Vasodilatador**					
Não	185 (90,7)	19 (9,3)	1	1	
Sim	123 (90,4)	13 (9,6)		1,03	0,52-2,01
**Vasodilatador coronariano**					
Não	302 (90,7)	31 (9,3)	0,503	1	
Sim	6 (85,7)	1 (14,3)		1,53	0,24-9,71

*RP = Razões de Prevalência; ^†^IC = Intervalo de Confiança. ^‡^Salário mínimo, Brasil, 2020 = R$ 1.039,00; ^§^Não foram encontrados os dados de 16 pacientes em relação a esse medicamento; ^ǁ^Versão 2 = Variável dicotomizada

Na Tabela 3, são apresentados os resultados do modelo de Cox, sendo possível observar que o aumento de uma unidade no escore do comportamento de autocuidado leva a um aumento de 8% na prevalência de não adesão do indivíduo; aqueles com renda familiar superior a três salários mínimos têm prevalência de não adesão ao tratamento igual a 3,5% da prevalência entre aqueles com até um salário mínimo e indivíduos que ingerem bebida alcoólica e que possuem depressão têm prevalências de não adesão 3,493 e 3,695 vezes maiores, respectivamente, do que aqueles que não apresentam tais antecedentes. 

**Tabela 3:** Resultados do modelo de Cox para adesão ao tratamento medicamentoso (n = 340). São Paulo, SP, Brasil, 2018-2020

	Modelo de Cox*			
	VIF^†^	RP^‡^	IC^§^ 95%	p-valor
Asma: Sim	1,539	2,311	0,767-6,963	0,137
Classe Funcional da *New York Association*	1,583	1,192	0,571-2,488	0,641
Depressão: Sim	1,426	3,695	1,538-8,876	0,003
Escolaridade	1,781	1,007	0,913-1,111	0,884
Escore da escala de autocuidado	1,599	1,080	1,029-1,133	0,002
Estado civil: sem companheiro	3,044	0,586	0,225-1,528	0,274
Idade	1,680	0,999	0,966-1,034	0,963
Ingestão de bebida alcoólica: Sim	1,373	3,493	1,150-10,604	0,027
Número de tomadas de medicamentos	2,076	0,992	0,876-1,122	0,893
Renda familiar: de um até três salários mínimos	2,269	0,270	0,071-1,024	0,054
Renda familiar: mais de três salários mínimos	2,269	0,035	0,004-0,328	0,003
Tempo da doença em anos	1,963	1,011	0,982-1,042	0,453

*Cox = Modelo de Cox; ^†^VIF= *Variance Inflation Factor*; ^‡^RP = Razão de Prevalência; ^§^IC = Intervalo de Confiança

## Discussão

A adesão ao tratamento farmacológico pode ser influenciada por diversos fatores comportamentais, sociais e econômicos. Esses fatores precisam ser monitorados para o sucesso do tratamento^30^.

Nesta pesquisa, observou-se que a maioria dos participantes era aderente ao tratamento medicamentoso, o que corrobora os achados da literatura. Estudo transversal realizado em São Paulo, com 100 pacientes com IC, mostrou que mais da metade dos participantes era aderente ou tinha moderada adesão^31^. Resultados de outro estudo realizado na Tailândia, que analisou 180 pacientes com IC, evidenciaram que 11,7% dos participantes apresentavam baixa adesão ao tratamento medicamentoso^32^. 

O resultado da adesão ao tratamento medicamentoso da maior parte dos participantes pode estar relacionado ao fato de que a IC é uma doença crônica, em que o indivíduo precisa se adequar aos novos hábitos de vida, assim como ao uso de muitos medicamentos de maneira contínua, para evitar a descompensação da doença e, consequentemente, não prejudicar sua rotina e sua qualidade de vida^32^.

Apesar de a maioria dos pacientes ser aderente ao tratamento farmacológico, foi possível identificar, no presente estudo, quatro fatores que se relacionaram à não adesão. A associação de um pior comportamento de autocuidado com a não adesão já era esperada, uma vez que, de acordo com a Organização Mundial de Saúde (OMS), o autocuidado é definido como “a capacidade de indivíduos, famílias e comunidades de promover a saúde, prevenir doenças, manter-se saudável e lidar com doenças e deficiências com ou sem o apoio de um profissional de saúde”^33^. Ainda, o uso correto dos medicamentos é um dos componentes do autocuidado^30^. A literatura reporta o autocuidado como um cardioprotetor, sendo complementar aos tratamentos farmacológicos e clínicos que podem retardar a progressão da IC e de seus resultados indesejáveis, como a descompensação clínica e readmissões^34^. Corroborando os achados do presente estudo, os resultados de uma pesquisa realizada no oeste da Etiópia, com 424 participantes com insuficiência cardíaca crônica, evidenciaram que mais da metade dos participantes aderia ao tratamento medicamentoso, além do fato de que os que apresentavam adequada adesão eram mais propensos a ter melhores comportamentos de autocuidado (OR=4,214; IC95%=2,725-6,515; p<0,001)^35^.

Baixa renda familiar também tem sido apontada na literatura como um dos fatores para a não adesão ao tratamento medicamentoso. Estudo realizado com 142.577 indivíduos com doenças cardiovasculares crônicas, cujo objetivo foi identificar os fatores sociodemográficos associados à não adesão ao tratamento medicamentoso, verificou que a baixa renda relacionou-se com a não adesão ao tratamento medicamentoso (OR=3,57; IC 95%=2,11-6,02)^36^. Um dos motivos dessa relação é a falta de dinheiro para adquirir os medicamentos não disponíveis no Sistema Único de Saúde, bem como para lidar com os custos relacionados ao acesso aos serviços de saúde. 

Ainda no presente estudo, outras duas variáveis relacionaram-se com a não adesão ao tratamento farmacológico: presença de depressão e uso de bebida alcoólica. A depressão tem sido associada à diminuição da adesão ao tratamento medicamentoso e da capacidade e/ou interesse em realizar o autocuidado, implicando a redução da qualidade de vida, maiores gastos relacionados aos serviços de saúde e respectivos aumentos das taxas de mortalidade^37^ ^)-(^ ^38^. Quanto ao uso de bebida alcoólica, estudo sugere que o álcool pode não estar diretamente relacionado à não adesão ao tratamento farmacológico, mas sim ao fato de que o seu uso desencadeia outros problemas de saúde física e mental que pioram a qualidade de sono e constituem fatores associados à não adesão^39^. Outras hipóteses dessa relação são: os pacientes podem se esquecer de tomar os medicamentos em razão dos efeitos do álcool^40^; eles têm medo de possíveis efeitos colaterais dos medicamentos, devido ao uso concomitante de bebida alcoólica; há falta de dinheiro para adquirir os medicamentos em razão do consumo do álcool^40^.

Este estudo tem limitações que precisam ser levadas em consideração: por ser um estudo de corte transversal, não há a possibilidade de estabelecimento de relações causais; por ter sido realizado em um ambulatório de um único centro, os resultados encontrados não podem ser generalizados para indivíduos com características distintas da nossa amostra e/ou hospitalizados; o instrumento utilizado não foi submetido à análise das propriedades psicométricas em estudos anteriores para esta amostra e, neste estudo, a consistência interna do instrumento utilizado para analisar a adesão ao tratamento foi aceitável. Dessa forma, estudos com amostras maiores que avaliem o desempenho e as propriedades psicométricas da MAT em indivíduos com IC ainda são necessários.

Apesar das limitações apontadas, os resultados obtidos no presente estudo têm implicações relevantes para a prática clínica. Torna-se importante reconhecer que indivíduos com IC que apresentam pior comportamento de autocuidado, menor renda familiar, depressão e que ingerem bebida alcoólica são mais propensos a apresentar não adesão ao tratamento medicamentoso. Portanto, indivíduos com essas características necessitam ser mais bem gerenciados em sua trajetória clínica. Sendo assim, o enfermeiro deve conhecer esses fatores para planejar as suas ações. Ressalta-se que o enfermeiro é um dos profissionais que mais contribuem para a educação em saúde, principalmente em indivíduos com doenças crônicas, como a IC. Dessa forma, os achados deste estudo poderão contribuir substancialmente para o direcionamento de intervenções, a fim de se alcançarem os melhores resultados de indivíduos que poderão apresentar as mesmas características clínicas e sociodemográficas que as encontradas. 

Portanto, considerando a escassez de literatura nacional, reforça-se a necessidade de estudos que analisem e identifiquem intervenções eficazes para a melhora da adesão ao tratamento medicamentoso, a fim de implementá-las na prática clínica, com o intuito de melhorar a qualidade de vida de pacientes com IC e reduzir readmissões e óbitos.

## Conclusão

A maioria dos participantes foi aderente ao tratamento farmacológico. Os fatores relacionados à não adesão ao tratamento farmacológico foram: comportamento de autocuidado inadequado, menor renda familiar, depressão e ingestão de bebida alcoólica. 

# Translation: Factores asociados a la no adhesión al tratamiento farmacológico de pacientes con insuficiencia cardíaca*

**DOI:** 10.1590/1518-8345.6756.4301

**Authors:** Jannaína Gomes de Lima, Alba Lucia Bottura Leite de Barros, Juliana de Lima Lopes

**Affiliations:** 1 Universidade Federal de São Paulo, Escola Paulista de Enfermagem, São Paulo, SP, Brasil.

**Keywords:** Cumplimiento de la Medicación, Cardiología, Cumplimiento y Adherencia al Tratamento, Atención de Enfermeira, Enfermería, Insuficiencia Cardíaca

## Abstract

**Objetivo:**

identificar los factores que contribuyen para la no adhesión al tratamiento farmacológico de pacientes con insuficiencia cardíaca.

**Método:**

estudio transversal y analítico que utilizó la escala de Medida de Adhesión al Tratamiento para evaluar la no adhesión al tratamiento farmacológico. Las variables independientes fueron recolectadas utilizando la *European Heart Failure Self-care Behavior Scale* y un instrumento elaborado por los autores, basado en estudio anterior. Fueron utilizadas pruebas estadísticas para el análisis de los datos, siendo considerados significativos los valores de p≤0,05.

**Resultados:**

la muestra estuvo compuesta por 340 pacientes; de esos, 9,4% fueron clasificados como no adherentes. Los resultados del análisis múltiple mostraron que el aumento de una unidad en el puntaje de autocuidado lleva a un aumento de 8% en la prevalencia de la no adhesión del individuo; pacientes con renta familiar superior a tres salarios mínimos tienen prevalencia de no adhesión al tratamiento igual a 3,5% de aquellos que reciben hasta un salario mínimo; individuos que ingieren bebida alcohólica y sufren de depresión, tienen prevalencias de no adhesión de 3,49 y 3,69 veces mayores, respectivamente, que aquellos que no tienen esos antecedentes.

**Conclusión:**

la no adhesión al tratamiento farmacológico se relacionó con comportamientos de autocuidado, renta familiar, depresión e ingestión de bebida alcohólica.

## Puntos destacados

**(1)** La adhesión al tratamiento farmacológico se relacionó con el comportamiento del autocuidado.

**(2)** Pacientes con renta familiar mayor que tres salarios tienen menor propensión a la no adhesión.

**(3)** Otra asociación con la no adhesión al tratamiento farmacológico fue la depresión.

## Introducción

Las enfermedades crónicas no transmisibles (ECNT) son responsables por elevadas tasas de morbilidad y mortalidad en todo el mundo^1^. Entre esas enfermedades, se incluye la insuficiencia cardíaca (IC), definida como un síndrome clínico complejo de carácter sistémico, que ocasiona disfunción cardíaca e inadecuado suministro sanguíneo para atender a las necesidades metabólicas del organismo^2^. 

En Brasil, en el año 2021, fue constatado que 31.336 individuos murieron como consecuencia de la IC^3^ - esa enfermedad fue responsable por un costo de 1,5 billones de reales entre enero de 2017 y diciembre de ese año - que la transforma en un problema de salud pública^4^. A su vez, en Estados Unidos de América entre 2015 y 2018, se constató la existencia de aproximadamente seis millones de habitantes con edad mayor o igual a 20 años con IC^5^, siendo que se estima en aproximadamente 46% de casos en los 15 años siguientes, totalizando más de ocho millones de individuos con IC hasta 2030^6^.

El tratamiento de la IC está compuesto por medidas farmacológicas y no farmacológicas. En muchos casos, ese tratamiento es complejo^7^. El tratamiento farmacológico tiene como finalidad amenizar los síntomas y reducir la morbilidad y las tasas de readmisiones y de muertes provenientes de la IC^7^. A su vez, el tratamiento no farmacológico se enfoca en la actividad física, adhesión al control de la ingestión de líquidos y sodio, cuidados con la alimentación, cesación del tabaco, interrupción del uso de alcohol, vacunación y monitorización del peso y de las señales y síntomas de la IC^2^.

A pesar de su importancia, la adhesión a las recomendaciones de autoadministración de la salud está por debajo de lo ideal, lo que aumenta el riesgo de mortalidad y hospitalizaciones^8^. Un estudio multicéntrico realizado en tres centros brasileños, denominado EMBRACE, mostró que la mala adhesión al tratamiento fue el principal motivo de descompensación de la enfermedad, representando 55% de los casos. A su vez, los que refirieron uso irregular, en la última semana, presentaron un riesgo 22% mayor de internación por mala adhesión^9^. Una investigación desarrollada en Italia analizó el efecto de la adhesión al tratamiento con medicamentos en la mortalidad y en la readmisión de pacientes con IC. Los datos de esa investigación fueron extraídos de un banco de datos que analizó 100.785 pacientes, que fueron agrupados con base en el número de clases de medicamentos prescritos (un, dos o tres). Los resultados mostraron que hubo reducción de 15% de readmisiones (OR=0,851; IC95%=0,821-0,882; p<0,0000) en pacientes adherentes a una clase de medicamentos y de 29% en pacientes adherentes a tres clases (OR=0,706; IC95%=0,651-0,767; p<0,0000). Además, hubo reducción de 28% en la mortalidad (OR=0,722; IC95%=0,691-0,755) de participantes adherentes a una clase de medicamentos y de 18% en los pacientes adherentes a tres clases (OR=0,818; IC95%=0,742-0,9; p<0,0000)^10^. Otro estudio realizado en 47 hospitales de siete países del Oriente Médio, tuvo como objetivo identificar los factores que contribuyen para la readmisión y la mortalidad de pacientes con IC. Los resultados encontrados apuntaron que la no adhesión a la dieta y no adhesión a los medicamentos son factores precipitantes y significativos en la readmisión y en la mortalidad (p<0,001)^11^.

Delante de este contexto, la implementación de programas de manejo de la enfermedad es muy importante^12^. Una revisión sistemática con metaanálisis evidenció que intervenciones educativas, consultas telefónicas y visitas domiciliares, contribuyen para la mejoría del resultado de los pacientes con IC^13^. Entre tanto, es necesario identificar los factores que contribuyen para la no adhesión, de modo que los enfermeros puedan direccionar sus intervenciones.

Existen diversos factores que pueden impactar en la adhesión al tratamiento. Una investigación brasileña realizada en Rio Grande del Sur, cuyo objetivo era analizar los factores asociados a la mala adhesión al tratamiento, presentó en sus resultados que la no adhesión estaba relacionada con: la edad avanzada; tener tres o más morbilidades; poseer incapacidad instrumental para la vida diaria; tomar tres o más medicamentos; no tener plan de salud y, tener que comprar todos o parte de los medicamentos^14^. Otro estudio nacional realizado en João Pessoa (Paraíba), con 50 pacientes, evidenció que los del sexo masculino o, con clase funcional III y con más de una comorbilidad asociada a la IC, demostraron menores puntajes de adhesión^15^.

Considerando: a) la importancia de identificar los factores asociados a la no adhesión al tratamiento con medicamentos, de pacientes con IC, para la planificación de la asistencia e implementación de intervenciones y b) la escasez de publicaciones en Brasil sobre el tema, surgió la siguiente pregunta de investigación: ¿Cuáles son los factores que se relacionan con la no adhesión al tratamiento farmacológico de pacientes con IC? De esa forma, el objetivo de este estudio fue identificar los factores que contribuyen para la no adhesión al tratamiento farmacológico de pacientes con insuficiencia cardíaca. 

## Método

### Tipo del estudio

Se trata de un estudio transversal y analítico. Para el desarrollo de esta sección, utilizamos las directrices recomendadas por el *Strengthening the Reporting of Observational Studies in Epidemiology* (STROBE)^16^.

### Local del estudio

El estudio fue realizado en el Ambulatorio de Miocardiopatía de un hospital público del estado de São Paulo.

### Período

La recolección de datos ocurrió en el período de 2018 a 2020.

### Población

Los participantes de la investigación fueron pacientes con diagnóstico médico de IC atendidos en el Ambulatorio de Miocardiopatía de un hospital público del estado de São Paulo.

### Criterios de inclusión y exclusión

Pacientes con IC, mayores de 18 años, sin déficit visual, auditivo y/o cognitivo y diagnosticados por el equipo médico. 

### Definición de la muestra

El tamaño de la muestra fue determinado por un estudio piloto con 21 pacientes, realizado en el período de 5 a 26 de abril de 2018. La muestra del test piloto fue por conveniencia, insiriendo los pacientes que se presentaron al servicio en ese período. El cálculo del tamaño de la muestra fue basado en el Coeficiente de Correlación de Spearman entre la adhesión al tratamiento con medicamentos y el tiempo de enfermedad (r= -0,162), con nivel de significación de 5% y poder de test de 80%. La elección del tiempo de enfermedad para el cálculo estuvo basada en estudios que muestran que el tiempo de enfermedad es un importante factor que contribuye para la adhesión al tratamiento^17^ ^)-(^ ^18^. A partir de ese cálculo, se obtuvo una muestra mínima de 297 participantes. Entre tanto, debido a posibles abandonos, fue aumentado el tamaño de la muestra en 10%, o sea, 340 pacientes. Para realizar ese cálculo, utilizamos la siguiente fórmula: $N={\left[\left(Z_{\alpha }+Z_{\beta }\right)+C\right]}^{2}+3$ , donde: $C=0.5\times \ln\left[\left(1+r\right)/\left(1-r\right)\right]$ , r = coeficiente de correlación esperado, N = Número total de sujetos necesarios, α = Nivel de significación y β = 1 - Poder del test^19^. 

### Variables del estudio

La variable dependiente del estudio fue la adhesión al tratamiento con medicamentos, obtenida por la escala de Medida de Adhesión al Tratamiento (MAT)^20^. Las variables independientes fueron seleccionadas de acuerdo con estudios de la literatura e investigación anterior^21^. En seguida, fueron divididas en sociodemográficas (edad, raza, sexo, religión, estado civil, renta familiar e individual, número de personas dependientes de la renta, vínculo de empleo y escolaridad) y clínicas (tiempo de enfermedad, número y nombre de los medicamentos prescritos, número de ingestión de los medicamentos, presencia de otras comorbilidades, tabaquismo, sedentarismo, ingestión de bebida alcohólica, clase funcional de la *New York Heart Asociation* y estadiamento de la enfermedad). Otra variable independiente investigada fue el comportamiento del autocuidado, obtenido por medio de la versión brasileña de la *European Heart Failure Self-care Behavior Scale* - EHFScBS^22^. Esas variables fueron obtenidas por medio de entrevista y/o consulta a la ficha médica del paciente.

### Procedimientos para la recolección de datos

Los potenciales participantes del estudio fueron identificados a partir de la agenda de consultas del Ambulatorio de Miocardiopatía. Los pacientes que atendieron a los criterios de inclusión fueron consultados personalmente, considerando el deseo de participar de la investigación voluntariamente. Aquellos que aceptaron firmaron el Término de Consentimiento Libre e Informado. Posteriormente, fue llenado el instrumento que contenía las variables sociodemográficas y clínicas. La adhesión al tratamiento con medicamentos fue evaluada por medio de la escala MAT ^(^ ^20^. El comportamiento de autocuidado fue obtenido por medio de la versión brasileña de la EHFScBS^22^.

### Instrumentos para la recolección de datos

El instrumento que contenía las variables sociodemográficas y clínicas fue elaborado por los autores de este estudio y basado en estudio anterior^21^.

La adhesión al tratamiento con medicamentos fue evaluada por medio de la escala MAT^20^, después de obtener la autorización de los autores de la escala original. La escala original fue desarrollada en Portugal y presenta un valor de alfa de Cronbach de 0,73^20^. En Brasil, esa escala fue traducida y validada para el idioma portugués en individuos con trastorno mental^23^ y con diabetes mellitus^24^. 

Ese cuestionario es formado por siete ítems y por una escala del tipo Likert de seis puntos que varía de 1 (siempre) a 6 (nunca). El nivel de adhesión es obtenido sumando el valor de cada ítem, y dividiéndolo por el número total de ítems. Así, los individuos que obtienen puntajes igual o superiores a 5 son considerados como adherentes^20^ ^),(^ ^23^ ^)-(^ ^24^.

El comportamiento de autocuidado fue obtenido por medio de la versión brasileña de la EHFScBS^22^. La escala original fue desarrollada y validada en 2003, por un grupo de investigadores de Holanda. El alfa de Cronbach varió de 0,79 a 0,92^25^. Fue traducida y validada en Brasil en 2012, cuando fueron realizadas las etapas de traducción, síntesis, retrotraducción, revisión por Comité de Especialistas, pretest, evaluación de consistencia interna (alfa de Cronbach) y reproductibilidad por medio del pretest y postest. El alfa de Cronbach varió de 0,61 a 0,70^22^. La EHFScBS posee cinco dominios y doce preguntas relacionadas al comportamiento del autocuidado. Las respuestas para cada ítem varían de 1 (yo concuerdo totalmente) a 5 (yo discuerdo totalmente). La puntuación varía de 12 a 60 puntos, en que 12 es el mejor autocuidado posible y 60 es el peor autocuidado^22^ ^),(^ ^25^.

### Tratamiento y análisis de los datos

Las variables cuantitativas fueron descritas por la media y desviación estándar, o por la mediana y cuartiles; las cualitativas fueron descritas por la frecuencia absoluta y el porcentaje. La adhesión al tratamiento farmacológico fue evaluada considerando dos categorías: adherente y no adherente. Para verificar la asociación de la adhesión al tratamiento farmacológico con las variables independientes cuantitativas, fue utilizado el test de Mann-Whitney. Para la asociación con las variables independientes categóricas, se utilizó el test exacto de Fisher y la medida de Razón de Prevalencia (RP). Para evaluar la asociación conjunta entre diferentes variables independientes y el resultado adhesión al tratamiento farmacológico, fue utilizado el modelo múltiple de Cox, con tiempos constantes y variancia robusta.

Las variables que obtuvieron un p-valor menor que 0,10 en el análisis bivariado y las de interés clínico para los investigadores, fueron incluidas en el análisis múltiple. Para evaluar se existían problemas de multicolinearidad, entre las variables predictoras, fue calculada la métrica *Variance Inflation Factor* (VIF), que indicó que no había fuerte correlación entre esas variables. Valores de VIF inferiores a 5 fueron considerados como corte para la clasificación de la existencia o no de multicolinearidad^26^. Fue utilizado el *software* estadístico R, versión 4.0^27^, para los análisis estadísticos, siendo consideradas estadísticamente relevantes los que obtuvieron valor de p≤0,05. La consistencia interna del instrumento fue medida por el coeficiente alfa de Cronbach, siendo considerados aceptables valores arriba de 0,60^28^.

### Aspectos éticos

El proyecto de investigación fue sometido al Comité de Ética en Investigación del Hospital São Paulo, siendo aprobado en el día 21 de marzo de 2018, con la Decisión nº 2.555.873. Todos los participantes firmaron el Término de Consentimiento Libre e Informado, atendiendo a la Resolución n.º 466/2012 del Consejo Nacional de Salud^29^.

## Resultados

Fueron recolectados los datos de 340 pacientes, con edad media de 58,1±12,9 años, que poseían en media 7,6±4,4 años de estudios. Al analizar el tiempo de enfermedad y la cantidad de medicamentos utilizados, se observó que los participantes presentaban, en media, 11,8±10,3 años de tiempo de la enfermedad y utilizaban en media 6±2,2 medicamentos. La mayoría consistía en individuos del sexo masculino (51,8%), casados o en concubinato (56,8%) y de la religión católica (58,6%), siendo predominantemente pardos (43,78%) y blancos (43,2%). Se observó que la mayoría no poseía vínculo de empleo (63,2%), con renta familiar entre uno y tres salarios mínimos (75,6%), seguida de más de tres hasta cinco salarios mínimos (11,8%).

Los medicamentos más utilizados fueron los betabloqueadores (n=298; 87,6%), diuréticos (n=269;79,1%), antagonistas de los receptores del mineralocorticosteroide (n=236; 69,4%), estatina (n= 183; 53,8%) e inhibidor de la enzima conversora de la angiotensina o bloqueador de la angiotensina 2 (n=180; 52,9%).

Los resultados del alfa de Cronbach indicaron una confiabilidad aceptable del instrumento (α=0,65).

En el análisis bivariado, las variables que se relacionaron con la adhesión al tratamiento fueron: comportamiento de autocuidado (Tabla 1), renta familiar (Tabla 2), depresión (Tabla 2) y uso de antagonistas de los receptores de mineralocorticoides (Tabla 2). 

**Tabla 1:** Relación de las variables cuantitativas con la adhesión al tratamiento farmacológico de acuerdo con el test de Mann-Whitney (n = 340). São Paulo, SP, Brasil, 2018-2020

Variables	Adherente (n=308)			No adherente (n=32)			p-valor
	Mediana	C25*	C75^†^	Mediana	C25*	C75^†^	
Escolaridad	8	4	11	7	4,75	10	0,423
Puntaje del autocuidado	23	19	28	26,5	22	34,7	0,001
Edad (años completos)	60	50	68	58,5	42	66,5	0,471
Número de personas dependientes de la renta	2	2	3	3	2	4	0,635
Número de ingestión de medicamentos	9	7	11	9	6	11	0,335
Tiempo de la enfermedad en años	8	3	17	10	3,75	18,7	0,200

*C25 = Cuartiles 25%; ^†^C75 = Cuartiles 75%

**Tabla 2:** Relación de las variables sociodemográficas cualitativas con la adhesión al tratamiento farmacológico de acuerdo con el test exacto de Fisher (n = 340). São Paulo, SP, Brasil, 2018-2020

	Adherente (n=308)	No adherente (n=32)	p-valor	RP*	IC^†^ 95%
	n (%)	n (%)			
**Estado civil**					
Casado/concubinato	175 (90,7)	18 (9,3)	0,96	1	
Divorciado	25 (92,6)	2 (7,4)		0,79	0,19-3,23
Soltero	69 (90,8)	7 (9,2)		0,99	0,43-2,27
Viudo	39 (88,6)	5 (11,4)		1,22	0,48-3,10
**Estado civil - versión 2** ^ǁ^					
Casado/concubinato	175 (90,7)	18 (9,3)	1,00	1	
Otros	133 (90,5)	14 (9,5)		1,02	0,52-1,98
**Raza**					
Blanca o mestiza	134 (91,2)	13 (8,8)	0,843	1	
Negra	39 (88,6)	5 (11,4)		1,28	0,48-3,41
Parda	135 (90,6)	14 (9,4)		1,06	0,52-2,18
**Religión**					
Católico	182 (91,5)	17 (8,5)	0,445	1	
Evangélico y otras religiones	111 (90,3)	12 (9,7)		1,14	0,56-2,30
Sin religión	15 (83,3)	3 (16,7)		1,95	0,63-6,03
**Renta familiar** ^‡^					
Menos de un salario mínimo	9 (75)	3 (25)	0,007	1	
De uno hasta tres salarios mínimos	229 (89,1)	28 (10,9)		0,44	0,15-1,23
Más de tres salarios mínimos	69 (98,6)	1 (1,4)		0,05	0,01-0,51
No sabe	1 (100)	0 (0)		-	-
**Renta individual** ^‡^					
Menos de un salario mínimo	36 (90)	4 (10)	0,575	1	
Un salario mínimo o más	253 (90)	25 (10)		0,899	0,33-2,44
No trabaja (desempleado o del hogar)	19 (86,4)	3 (13,6)		1,364	0,33-5,55
**Sexo**					
Femenino	146 (89)	18 (11)	0,359	1	
Masculino	162 (92)	14 (8)		0,72	0,37-1,41
**Vínculo de empleo**					
Activo	90 (90,9)	9 (9,1)	1	1	
Del hogar	24 (92,3)	2 (7,7)		0,846	0,19-3,68
Inactivo	194 (90,2)	21 (9,8)		1,074	0,51-2,26
**Accidente vascular encefálico**					
No	251 (90,6)	26 (9,4)	1	1	
Si	57 (90,5)	6 (9,5)		1,015	0,44-2,36
**Arritmia**					
No	226 (91,1)	22 (8,9)	0,539	1	
Si	82 (89,1)	10 (10,9)		1,22	0,60-2,49
**Asma**					
No	295 (91,3)	28 (8,7)	0,064	1	
Si	13 (76,5)	4 (23,5)		2,71	1,07-6,86
**Bronquitis**					
No	298 (91,1)	29 (8,9)	0,113	1	
Si	10 (76,9)	3 (23,1)		2,60	0,91-7,45
**Clase funcional**					
I	71 (92,2)	6 (7,8)	0,832	1	
II	230 (89,8)	26 (10,2)		1,303	0,56-3,05
III	7 (100)	0 (0)		-	-
**Depresión**					
No	286 (92,6)	23 (7,4)	0,001	1	
Si	22 (71)	9 (29)		3,9	1,98-7,67
**Diabetes mellitus**					
No	209 (92,1)	18 (7,9)	0,236	1	
Si	99 (87,6)	14 (12,4)		1,562	0,81-3,02
**Enfermedad vascular periférica**					
No	232 (90,3)	25 (9,7)	0,831	1	
Si	76 (91,6)	7 (8,4)		0,87	0,39-1,93
**Enfisema pulmonar**					
No	300 (90,4)	32 (9,6)	1	1	
Si	8 (100)	0 (0)		-	-
**Estadiamento de la enfermedad**					
A	72 (92,3)	6 (7,7)	0,781	1	
B	228 (89,8)	26 (10,2)		1,331	0,57-3,11
C	8 (100)	0 (0)		-	-
**Hipercolesterolemia**					
No	181 (91,9)	16 (8,1)	0,353	1	
Si	127 (88,8)	16 (11,2)		1,38	0,71-2,66
**Hipertrigliceridemia**					
No	288 (90)	32 (10)	0,237	1	
Si	20 (100)	0 (0)		-	-
**Hipertensión arterial sistémica**					
No	87 (91,6)	8 (8,4)	0,837	1	
Si	221 (90,2)	24 (9,8)		1,163	0,54-2,49
**Ingestión de bebida alcohólica**					
No	295 (91,3)	28 (8,7)	0,064	1	
Si	13 (76,5)	4 (23,5)		2,714	1,07-6,86
**Insuficiencia renal crónica**					
No	227 (90,4)	24 (9,6)	1	1	
Si	81 (91)	8 (9)		0,94	0,44-2,01
**Sedentarismo**					
No	121 (90,3)	13 (9,7)	1	1	
Si	187 (90,8)	19 (9,2)		0,951	0,49-1,86
**Síndrome coronario agudo**					
No	213 (91)	21 (9)	0,691	1	
Si	95 (89,6)	11 (10,4)		1,156	0,58-2,31
**Síndrome de la inmunodeficiencia adquirida**					
No	307 (90,6)	32 (9,4)	1	1	
Si	1 (100)	0 (0)		-	-
**Tabaquismo**					
No	296 (90,5)	31 (9,5)	1	1	
Si	12 (92,3)	1 (7,7)		0,811	0,12-5,49
**Antagonistas de los receptores de mineralocorticoides** ^§^					
No	74 (84,1)	14 (15,9)	0,017	1	
Si	220 (93,2)	16 (6,8)		0,43	0,22-0,84
**Antiagregante plaquetario**					
No	211 (88,7)	27 (11,3)	0,069	1	
Si	97 (95,1)	5 (4,9)		0,43	0,17-1,09
**Antiarrítmico**					
No	299 (90,9)	30 (9,1)	0,277	1	
Si	9 (81,8)	2 (18,2)		1,99	0,54-7,31
**Anticoagulante**					
No	210 (90,5)	22 (9,5)	1	1	
Si	98 (90,7)	10 (9,3)		0,98	0,48-1,99
**Betabloqueador**					
No	35 (83,3)	7 (16,7)	0,093	1	
Si	273 (91,6)	25 (8,4)		0,50	0,23-1,09
**Bloqueador del canal de calcio**					
No	276 (91,4)	26 (8,6)	0,149	1	
Si	32 (84,2)	6 (15,8)		1,83	0,81-4,17
**Intoxicación por digitálicos**					
No	263 (90,4)	28 (9,6)	1	1	
Si	45 (91,8)	4 (8,2)		0,85	0,31-2,31
**Diurético**					
No	64 (90,1)	7 (9,9)	0,823	1	
Si	244 (90,7)	25 (9,3)		0,94	0,42-2,09
**Estatina**					
No	140 (89,2)	17 (10,8)	0,459	1	
Si	168 (91,8)	15 (8,2)		0,76	0,39-1,47
**Hipoglicemiante oral**					
No	235 (91,1)	23 (8,9)	0,664	1	
Si	73 (89)	9 (11)		1,23	0,59-2,55
**Bloqueador angiotensina 2, inhibidor de la enzima conversora de la angiotensina o ivabradina**					
No	144 (90)	16 (10)	0,853	1	
Si	164 (91,1)	16 (8,9)		0,89	0,46-1,72
**Insulina**					
No	292 (90,7)	30 (9,3)	0,682	1	
Si	16 (88,9)	2 (11,1)		1,19	0,31-4,60
**Vasodilatador**					
No	185 (90,7)	19 (9,3)	1	1	
Si	123 (90,4)	13 (9,6)		1,03	0,52-2,01
**Vasodilatador coronario**					
No	302 (90,7)	31 (9,3)	0,503	1	
Si	6 (85,7)	1 (14,3)		1,53	0,24-9,71

*RP = Razones de Prevalencia; ^†^IC = Intervalo de Confianza. ^‡^Salario mínimo, Brasil, 2020 = R$ 1.039,00; ^§^No fueron encontrados los datos de 16 pacientes en relación a ese medicamento; ^ǁ^ Versión 2 = Variable dicotomizada

En la Tabla 3, se presentan los resultados del modelo de Cox, en que es posible observar que el aumento de una unidad en el puntaje del comportamiento de autocuidado lleva a un aumento de 8% en la prevalencia de no adhesión del individuo; aquellos con renta familiar superior a tres salarios mínimos tienen prevalencia de no adhesión al tratamiento igual a 3,5% de la prevalencia entre aquellos con hasta un salario mínimo; individuos que ingieren bebida alcohólica y que poseen depresión tienen prevalencias de no adhesión 3,493 y 3,695 veces mayores, respectivamente, que aquellos que no presenten esos antecedentes. 

**Tabla 3:** Resultados del modelo de Cox para adhesión al tratamiento con medicamentos (n = 340). São Paulo, SP, Brasil, 2018-2020

	Modelo de Cox*			
	VIF^†^	RP^‡^	IC^§^ 95%	p-valor
Asma: Si	1,539	2,311	0,767-6,963	0,137
Clase Funcional de la *New York Association*	1,583	1,192	0,571-2,488	0,641
Depresión: Si	1,426	3,695	1,538-8,876	0,003
Escolaridad	1,781	1,007	0,913-1,111	0,884
Puntaje de la escala de autocuidado	1,599	1,080	1,029-1,133	0,002
Estado civil: sin compañero	3,044	0,586	0,225-1,528	0,274
Edad	1,680	0,999	0,966-1,034	0,963
Ingestión de bebida alcohólica: Si	1,373	3,493	1,150-10,604	0,027
Número de ingestión de medicamentos	2,076	0,992	0,876-1,122	0,893
Renta familiar: de uno hasta tres salarios mínimos	2,269	0,270	0,071-1,024	0,054
Renta familiar: más de tres salarios mínimos	2,269	0,035	0,004-0,328	0,003
Tiempo de la enfermedad en años	1,963	1,011	0,982-1,042	0,453

*Cox = Modelo de Cox; ^†^VIF = *Variance Inflation Factor*; ^‡^RP = Razón de Prevalencia; ^§^IC = Intervalo de Confianza

## Discusión

La adhesión al tratamiento farmacológico puede ser influenciada por diversos factores comportamentales, sociales y económicos. Esos factores deben ser monitorizados para obtener éxito en el tratamiento^30^.

En esta investigación, se observó que la mayoría de los participantes era adherente al tratamiento con medicamentos, lo que es corroborado en los resultados de la literatura. Un estudio transversal realizado en Sao Paulo, con 100 pacientes con IC, mostró que más de la mitad de los participantes era adherente o tenía moderada adhesión^31^. Los resultados de otro estudio realizado en Tailandia, que analizó 180 pacientes con IC, mostraron que 11,7% de los participantes presentaban baja adhesión al tratamiento con medicamentos^32^. 

El resultado de la adhesión al tratamiento con medicamentos, de la mayor parte de los participantes, puede estar relacionado a que la IC es una enfermedad crónica, que necesita que el individuo se adecue a los nuevos hábitos de vida, así como al uso de muchos medicamentos de manera continua, para evitar la descompensación de la enfermedad y, consecuentemente, no perjudicar su rutina y calidad de vida^32^.

A pesar de que la mayoría de los pacientes es adherente al tratamiento farmacológico, fue posible identificar, en este estudio, cuatro factores que se relacionaron a la no adhesión. La asociación de un peor comportamiento del autocuidado con la no adhesión, ya era esperada, considerando que, de acuerdo con la Organización Mundial de la Salud (OMS), el autocuidado es definido como “la capacidad de individuos, familias y comunidades de promover la salud, prevenir enfermedades, mantenerse saludable y lidiar con enfermedades y deficiencias con o sin el apoyo de un profesional de salud”^33^. Además, el uso correcto de los medicamentos es uno de los componentes del autocuidado^30^. La literatura define al autocuidado como un protector cardiológico, siendo complementario a los tratamientos farmacológicos y clínicos, que pueden retardar la progresión de la IC y sus resultados indeseables, como la descompensación clínica y readmisiones^34^. Corroborando los resultados de este estudio, los resultados de una investigación, realizada en el oeste de Etiopia, con 424 participantes con insuficiencia cardíaca crónica, evidenciaron que más de la mitad de los participantes adhirió al tratamiento con medicamentos; además, los que presentaban adecuada adhesión eran más propensos a obtener mejores comportamientos de autocuidado (OR=4,214; IC95%=2,725-6,515; p<0,001)^35^.

La baja renta familiar también ha sido apuntada en la literatura como uno de los factores para la no adhesión al tratamiento con medicamentos. Un estudio realizado con 142.577 individuos con enfermedades cardiovasculares crónicas, que tenía por objetivo identificar los factores sociodemográficos asociados a la no adhesión al tratamiento con medicamentos, verificó que la baja renta se relacionó con la no adhesión al tratamiento con medicamentos (OR=3,57; IC 95%=2,11-6,02)^36^. Uno de los motivos de esa relación es la falta de dinero para adquirir los medicamentos que no están disponibles en el Sistema Único de Salud, así como para lidiar con los costos relacionados al acceso a los servicios de salud. 

Además, en este estudio, otras dos variables se relacionaron con la no adhesión al tratamiento farmacológico: la presencia de depresión y el uso de bebida alcohólica. La depresión ha sido asociada a la disminución de la adhesión al tratamiento con medicamentos y a la capacidad y/o interés en realizar el autocuidado, lo que conduce a la reducción de la calidad de vida, mayores gastos relacionados a los servicios de salud y respectivos aumentos de las tasas de mortalidad^37^ ^)-(^ ^38^. En lo que se refiere al uso de bebida alcohólica, un estudio sugiere que el alcohol puede no estar directamente relacionado a la no adhesión al tratamiento farmacológico, pero si al hecho de que su uso desencadena otros problemas de salud física y mental, que empeoran la calidad del sueño; así, formando parte de factores asociados a la no adhesión^39^. Otras hipótesis de esa relación son: pacientes que pueden olvidarse de tomar los medicamentos debido a los efectos del alcohol^40^; los tienen miedo de posibles efectos colaterales de los medicamentos, debido al uso concomitante de bebida alcohólica y la falta de dinero para adquirir los medicamentos debido al gasto con consumo de alcohol^40^.

Este estudio tiene limitaciones que deben ser consideradas: 1) por ser un estudio de corte transversal, no existe la posibilidad de establecer relaciones causales; 2) porque se realizó en un ambulatorio de un único centro, los resultados encontrados no pueden ser generalizados para individuos con características distintas de nuestra muestra y/o para hospitalizados; 3) el instrumento utilizado no fue sometido al análisis de las propiedades psicométricas en estudios anteriores para esta muestra y, 4) en este estudio, la consistencia interna del instrumento utilizado para analizar la adhesión al tratamiento fue aceptable. Así, estudios con muestras mayores que avalúen el desempeño y las propiedades psicométricas de la MAT en individuos con IC todavía son necesarios.

A pesar de las limitaciones apuntadas, los resultados obtenidos en este estudio tienen implicaciones relevantes para la práctica clínica. Es importante reconocer que individuos con IC que presentan peor comportamiento de autocuidado, menor renta familiar, depresión y que ingieren bebida alcohólica, son más propensos a presentar la no adhesión al tratamiento con medicamentos. Por tanto, individuos con esas características necesitan ser mejor administrados en su trayectoria clínica. Luego, el enfermero debe conocer esos factores para planificar sus acciones. Se destaca que, el enfermero es uno de los profesionales que más contribuye para la educación en salud, principalmente en individuos con enfermedades crónicas, como a IC. De esa forma, los resultados de este estudio pueden contribuir substancialmente para el direccionamiento de intervenciones, que permitan alcanzar mejores resultados en individuos, que presenten las mismas características clínicas y sociodemográficas que las encontradas. 

Por tanto, considerando la escasez de literatura nacional, se refuerza la necesidad de realizar estudios que analicen e identifiquen intervenciones eficaces para la mejoría de la adhesión al tratamiento con medicamentos, con la finalidad de implementarlas en la práctica clínica y así mejorar la calidad de vida de pacientes con IC y reducir readmisiones y muertes.

## Conclusión

La mayoría de los participantes fue adherente al tratamiento farmacológico. Los factores relacionados a la no adhesión al tratamiento farmacológico fueron: comportamiento de autocuidado inadecuado, menor renta familiar, depresión e ingestión de bebida alcohólica.
